# Umbilical Venous Catheter Position: The Value of Acquiring a Lateral in Addition to a Frontal Chest Radiograph

**DOI:** 10.7759/cureus.46642

**Published:** 2023-10-07

**Authors:** Nawaf Al-Dajani, Horacio Osiovich, John Smyth, Angela Byrne, Douglas Jamieson, Gregor Kaczala

**Affiliations:** 1 Pediatric Department, King Abdulaziz University, Jeddah, SAU; 2 Pediatric Department, British Columbia Children & Women's Hospital, Vancouver, CAN; 3 Radiology, Children's Health Ireland, Dublin, IRL; 4 Radiology Department, British Columbia Children & Women's Hospital, Vancouver, CAN; 5 Pediatric Intensive Care Unit, Praxiszentrum am Bahnhof Bern, Bren, CHE

**Keywords:** umbilical venous catheters, central venous access, neonates, lateral chest radiographs, frontal radiographs

## Abstract

Introduction

Umbilical venous catheters (UVCs) are standardly used for central venous access in acutely sick neonates. Complications associated with UVCs include thrombosis, infection, diffuse intravascular coagulopathy, arrhythmia, tamponade, and liver injury, many of which are related to misplacement of the catheters. Therefore, this study aimed to institute a policy of obtaining lateral and frontal radiographs to improve the determination of the UVC position.

Methods

We retrospectively reviewed UVC placement from 132 radiographs. We compared interpretations by different reviewers of frontal versus frontal and lateral chest radiographs for the most accurate determination of the UVC position. The reviewers completed questionnaires indicating their assessment of the catheter tip position, as well as the appropriate catheter manipulation required for optimal positioning. Their assessment was derived from frontal chest radiographs followed by frontal plus lateral view radiographs a week later.

Results

The reviewers (junior neonatology fellow, senior neonatology fellow, pediatric radiology fellow, and senior pediatric radiologist) revised their assessment with regard to the UVC positioning between frontal and frontal plus lateral radiographs in 24.6%, 22.7%, 19.6%, and 15.9% of cases, respectively, and indicated that the lateral view was helpful in 18%, 13.6%, 19.6%, and 31% of the cases, respectively. UVCs were placed appropriately at the first attempt in only 13.6% of the cases.

Conclusion

Correct initial placement of a UVC is uncommon. A lateral radiograph is beneficial in determining the UVC position. Hence, we suggest the inclusion of a lateral view along with the frontal chest radiograph for the evaluation of the UVC position if real-time ultrasound cannot be performed before UVC usage.

## Introduction

Umbilical vessel catheterization is standardly used for central vascular access in sick neonates. An umbilical vein catheter (UVC) can be introduced through the umbilical vein and ductus venosus to access the inferior vena cava (IVC) to provide resuscitation and support to the sick neonates. UVCs can be used to infuse fluids, administer parenteral nutrition and medications, monitor central venous pressure, obtain blood samples for laboratory tests, and perform exchange transfusions [1-4].

The UVC insertion length is generally based on predetermined correlations between external body measurements (e.g., shoulder-umbilicus distance) and the regression equation based on birth weight [3,4]. Insertion of a UVC is usually performed blindly; subsequently, the actual position of the UVC is routinely checked on radiographs. The recommended position for the UVC tip is in the IVC, above the junction of the ductus venosus, or at the IVC and right atrium (RA) junction, which corresponds to thoracic vertebral bodies 7-9 [1,5-7]. The RA-IVC junction cannot be visualized on frontal chest radiographs; thus, the level of the diaphragm is used as a landmark for this location. However, this level is variable and affected by lung volume, phase of respiration, dome of the diaphragm, and angle of the X-ray beam. Direct visualization by echocardiography or ultrasonography is considered the most accurate method for determining the tip position of UVCs [6-10]. However, the expertise and equipment required for echocardiography or ultrasonography are not always readily available during the insertion of these catheters. Frontal chest radiography is the most common method used to determine the UVC tip position in many neonatal intensive care units (NICUs). However, it has been demonstrated to be unreliable in determining the location of the UVC tip [8]. A previous study reported that frontal chest radiographs have a sensitivity and specificity of 32% and 89%, respectively, in evaluating inappropriate UVC positioning while lateral chest radiographs have a sensitivity and specificity of 76% and 33%, respectively [8].

UVCs malpositioned above the diaphragm are associated with various complications, including cardiac arrhythmias, cardiac tamponade, thrombus formation, and perforation of the pulmonary veins [8,11-13]; UVCs malpositioned below the diaphragm are associated with liver necrosis, hematoma, and umbilical vein perforation [2,9,14,15]. Echocardiography and real-time ultrasonography have been used to determine the UVC position with more accuracy and certainty, although due to a lack of expertise and the necessity of urgent use of the UVC, plain radiography is used as a standard procedure in many NICUs worldwide [6,7,9-11]. Electrocardiography has also been used as an ancillary method to determine the position of the UVC based on heart rate changes [5].

To reduce adverse events and to improve our determination of the UVC position, we instituted a policy of performing routine lateral radiography, in addition to the standard frontal chest radiography. As this policy entailed added expense and radiation exposure, it was important to evaluate the benefit of lateral radiographs in determining the UVC position.

## Materials and methods

Study design

This is a retrospective study reviewing frontal and lateral chest and upper abdomen radiographs obtained to assess the placement of UVCs, from January 1 to December 31, 2004. Four reviewers (a junior and a senior neonatology fellow, a pediatric radiology fellow, and a senior pediatric radiologist) each independently recorded their assessment of the position of the UVC tip, as observed on a frontal radiograph (132 radiographs), using a standard questionnaire. This was followed by a review of the frontal radiograph combined with a lateral radiograph one week later. Additionally, the reviewers described the catheter manipulation required to correct the malpositioned catheter. The reviewers also rated the usefulness of the additional lateral radiograph at the second reading. The reviewers were blinded to patient names and were given a one-week interval between assessments to reduce case recognition. Each reviewer reassessed 10% of the cases one week after the initial assessment such that within-reviewer reproducibility could be examined. Appropriate catheter position was defined as the positioning of the UVC tip in the intrathoracic IVC (at the IVC-RA junction) or at the level of the diaphragm.

Statistical analyses

SPSS version 14.0 program (SPSS Inc., Chicago, IL) was used for statistical analyses. Cross-tabulations of observations for frontal versus frontal plus lateral radiographs were calculated for each of the questions. Agreement across categories was assessed using kappa statistics. The percentage of total agreement was extracted, and 95% confidence intervals (CIs) were estimated to provide a measure of precision. The intra-rater agreement between frontal and frontal/lateral views for each of the questions within each individual reviewer was measured via kappa statistics. The total agreement was presented as percentages and 95% CI.

## Results

A total of 132 pairs of radiographs were reviewed. UVCs were judged to be placed appropriately at the first attempt in only 18 patients (13.6%) of the cases (95% CI: 7.8, 19.5). In 48% of the cases, UVCs were located well below the diaphragm.

The reviewers revised their answers with regard to UVC positioning between frontal and frontal plus lateral radiographs as follows: the junior neonatology fellow in 32 radiographs (24.2%) (95% CI: 17.2, 32.0), senior neonatology fellow in 30 radiographs (22.7%) (95% CI: 15.6, 30.0), the radiology fellow in 26 radiographs (19.6%) (95% CI: 12.9, 26.5), and pediatric radiologist in 21 radiographs (15.9%) (95% CI: 9.7, 22.1) of the comparisons. The junior neonatology fellow, senior neonatology fellow, radiology fellow, and pediatric radiologist indicated that the lateral view was helpful in 24 (18%), 18 (13.6%), 26 (19.6%), and 41 (31%) of the cases, respectively (Table 1).

**Table 1:** Percentage of change in the assessment of UVC position after reviewing lateral radiographs UVC: umbilical venous catheter

Reviewer	% of change after reviewing the lateral view	% of helpfulness of the lateral view
Junior neonatology fellow	24.2	18
Senior neonatology fellow	22.7	13.6
Radiology fellow	19.6	19.6
Senior radiologist	15.9	31

The consistency between the interpretations of the junior neonatology fellow and those of the senior pediatric radiologist was 90%. When the UVC position was assessed as one of three categories (above the diaphragm, at the level of the diaphragm, or below the level of the diaphragm), the reviewers changed their answers in 18 (13.6%), 36 (27.2%), and 80 (60.6%) of the comparisons, respectively (Table 2).

**Table 2:** Impact of the UVC/diaphragm relationship on the assessment of UVC position in lateral radiographs UVC: umbilical venous catheter

UVC position in relation to the diaphragm	% of change in the assessment of the UVC position
UVC above the diaphragm	13.6 %
UVC at the diaphragm level	27.2%
UVC below the diaphragm level	60.6%

The overall reproducibility of interpretation was 98%. The individual reproducibility of interpretation was 100% for the senior pediatric radiologist and radiology fellow for both groups, 100% for the neonatology fellows for frontal plus lateral radiographs, and 92% for frontal radiographs alone. Kappa statistics between frontal and frontal plus lateral radiographs across the four reviewers ranged from 0.6 to 1.0 for the position of the tip, from 0.9 to 1.0 for the location, and from 0.7 to 1.0 for the action that should be taken.

For intra-rater reliability, kappa statistics were calculated between the readings of the same sample. For the four reviewers, kappa ranged from 0.68 to 1 for the frontal view and increased to 0.74 to 1.0 for the frontal plus lateral view.

## Discussion

The acquisition of lateral radiographs for UVC position assessment demonstrated a reasonable impact on decision-making, clearly benefitting the assessment of the reviewers who had different levels of expertise. Our study findings emphasized the helpfulness of lateral radiographs for position assessment, as well as for further management of UVCs.

Although the radiographs in our study were reviewed retrospectively, they approximated a prospective method and the reviewers were blinded to the clinical outcome. Additionally, the reviewers were blinded to the lateral views when evaluating the frontal view. To the best of our knowledge, the sample size of our cohort is probably the largest in the literature to date, and the findings of the reviewers were reproducible.

A few studies with a limited number of radiographs have highlighted the importance of lateral radiographs in measuring the catheter tip/diaphragm distance, although they have not evaluated the usefulness of lateral radiographs in assessing catheter tip placement [14,16]. To the best of our knowledge, this report is the largest study comparing frontal radiographs to both frontal and lateral radiographs for determining the UVC position.

The strength of our study is in the comparison of interpretations between junior and senior staff, including a senior pediatric radiologist. The junior reviewer was more likely to change their interpretation when presented with lateral radiographs, compared with the senior or radiology reviewers. Thus, the addition of lateral radiographs may be especially beneficial to junior staff members who are typically the first line on call. This will provide them with the opportunity to correct the catheter position before using UVCs. In addition, for the junior fellow, the lateral view demonstrated 100% reproducibility of the UVC position compared with 92% reproducibility for the frontal view alone. The study also demonstrated consistency between the junior fellow and radiologist in interpreting radiographs, with a 90% concordance regarding the assessment of the UVC tip position.

Of the UVCs placed in our unit, only 18 (13.6%) were placed in the desired appropriate position at the first attempt. This result is similar to the 23% reported in another published study (8). This suggests that appropriate position achievement is uncommon. Hence, the current practice of blind insertion with a predetermined length is inappropriate and exposes infants to potential risks.

UVCs were located below the diaphragm in 64 (48.4%) of the cases, indicating that the chance of accessing the ductus venosus and traversing it at the first attempt was only 51.6%. Thus, around half of the UVCs inserted had appropriate central venous access. All UVCs that gain access to the IVC have the potential to be positioned correctly. The higher malpositioned catheter tips could be carefully withdrawn to an appropriate length, as determined by the radiographs.

**Figure 1:**
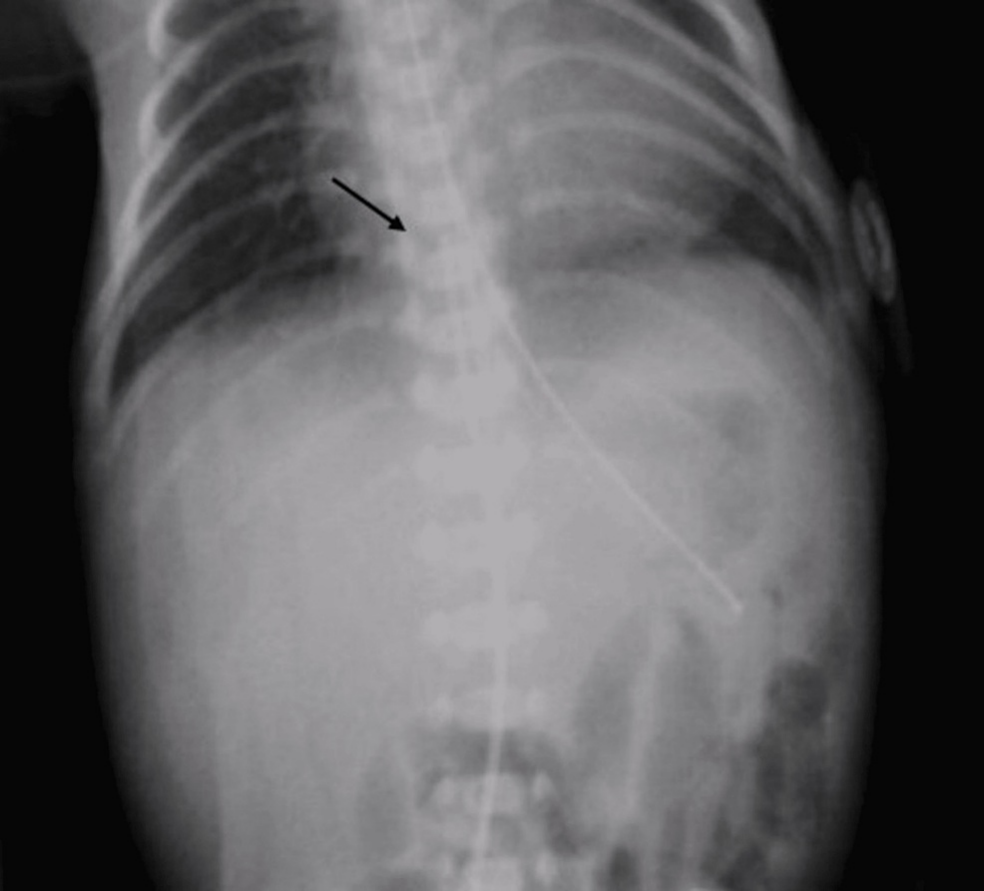
Frontal anteroposterior (AP) view showing the umbilical venous catheter (black arrow) in the right atrium in Case 1

**Figure 2:**
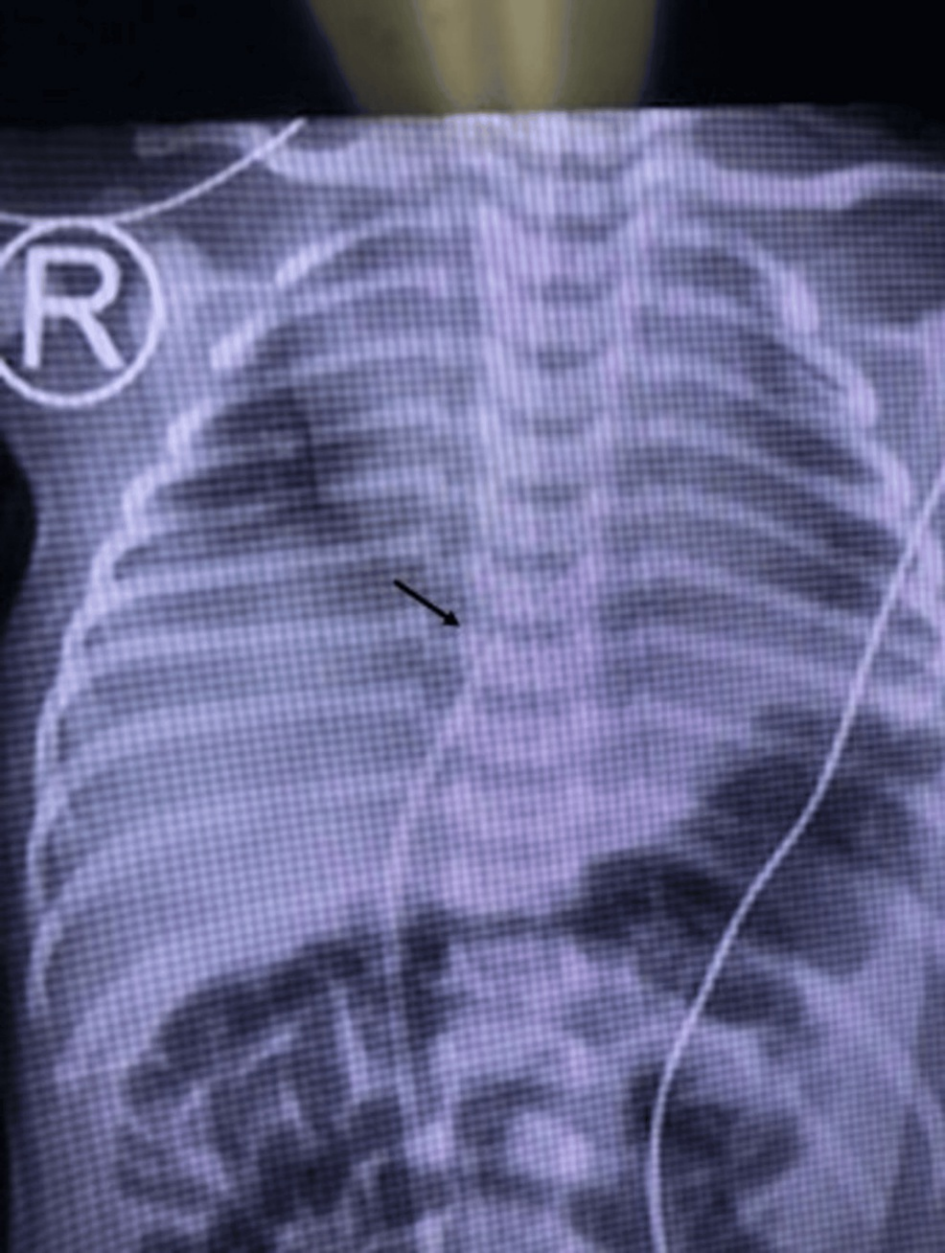
Frontal anteroposterior (AP) view showing the position of the umbilical venous catheter (black arrow) in the liver in Case 2

Frontal radiographs demonstrate the fairly straight course of a catheter and readily exhibit deviations into the left or right portal vein (Figures 1, 2).

**Figure 3:**
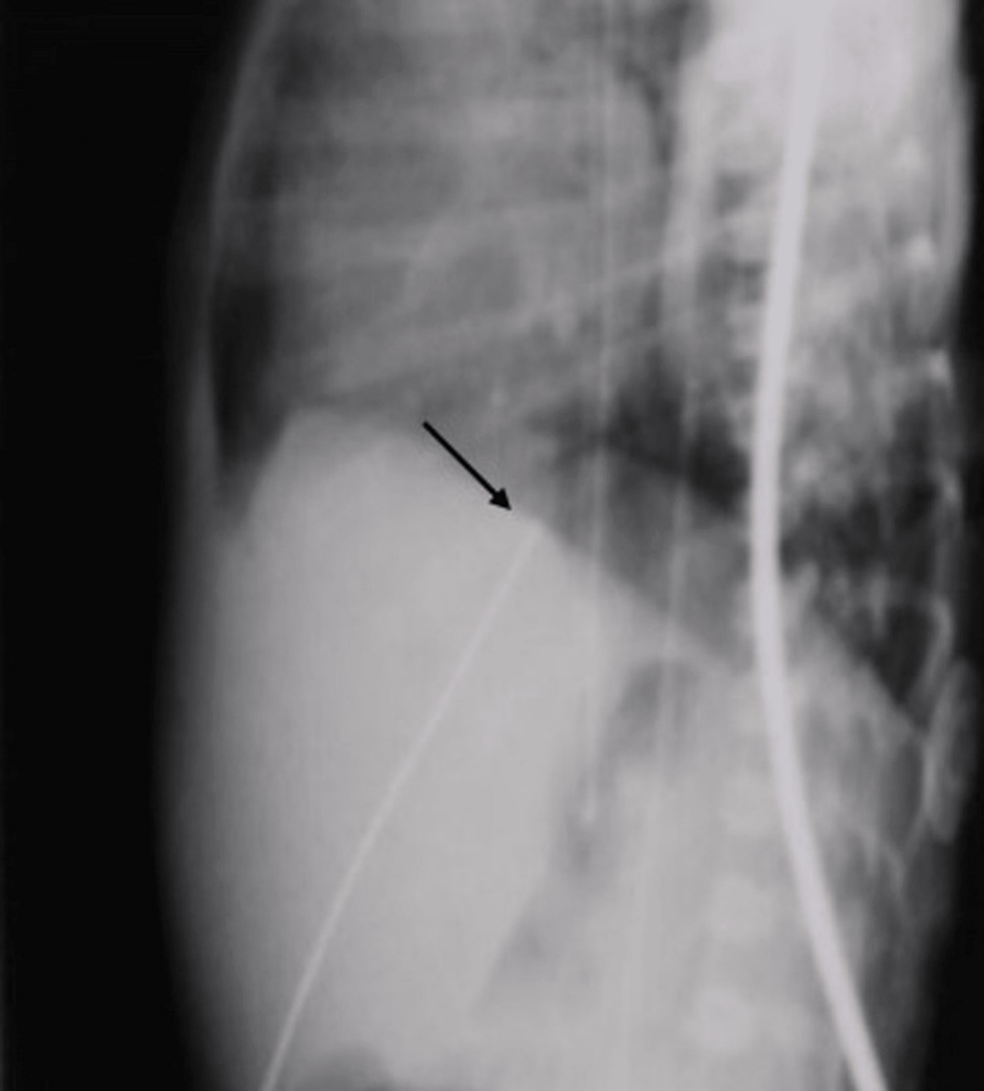
Lateral view showing the umbilical venous catheter (black arrow) perfectly positioned at the junction of the inferior vena cava and the right atrium at the level of the diaphragm in Case 1

**Figure 4:**
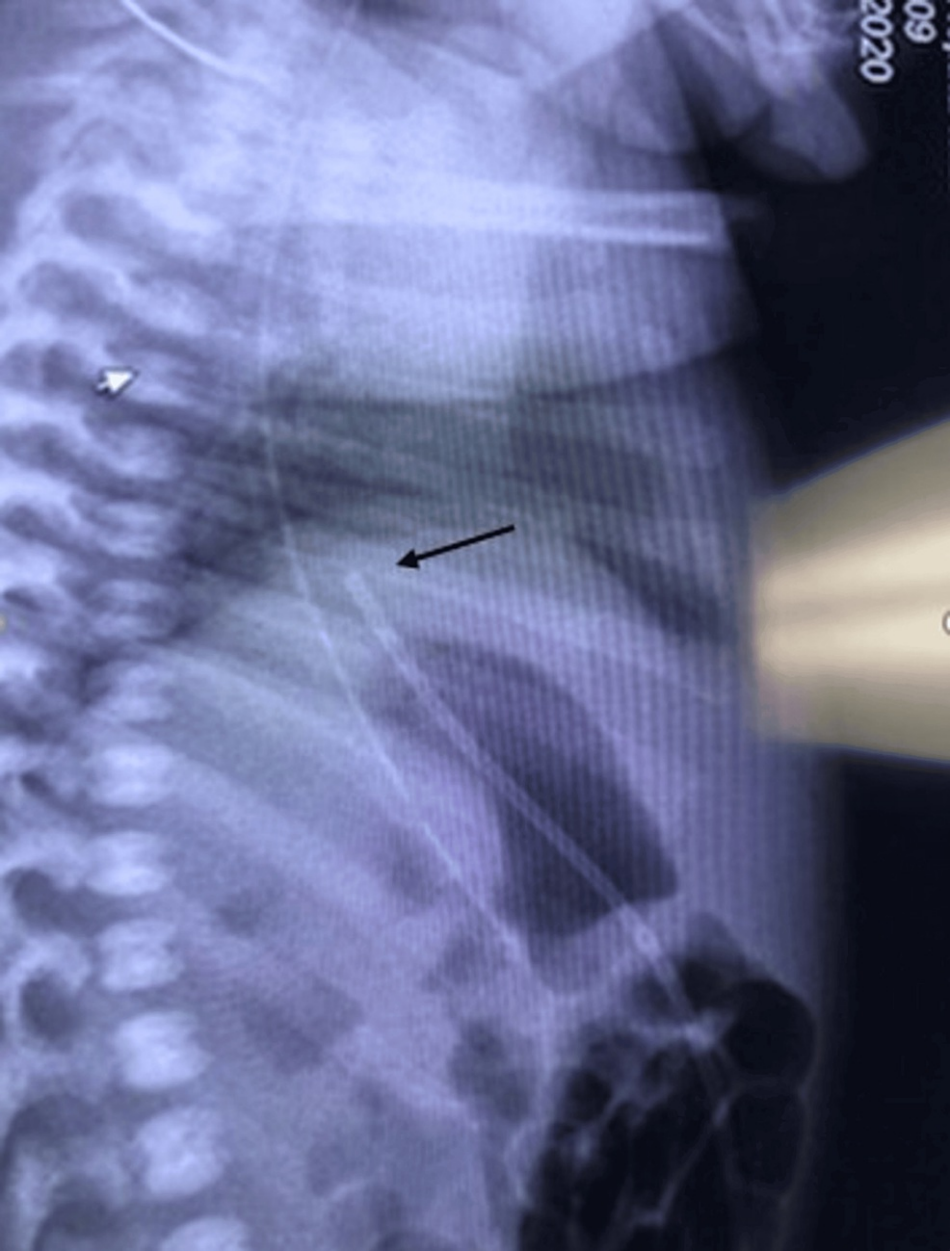
Lateral view showing the umbilical venous catheter (black arrow) at the junction of the inferior vena cava and right atrium in Case 2

In contrast, the lateral view demonstrates the characteristic S curve of the UVC, as it traverses the umbilical vein-portal vein-ductus venosus. Hence, anterior or posterior deviations into the hepatic parenchyma can be identified (Figures 3, 4). The lateral view also has the advantage of enabling reviewers to identify several anatomical landmarks such as the dome of the diaphragm, posterior costophrenic insertion of the diaphragm, IVC-RA junction, and edges of the liver. Our study demonstrated the lateral radiograph to be more helpful if the UVCs were placed below or at the level of the diaphragm and less helpful if the UVCs were placed above the diaphragm (frontal view in Figures 1, 2 versus lateral view in Figures 3, 4).

This was presumably because the anatomical landmarks for high catheter tip positionings were less critical to identify (Table 2). The reviewers found the lateral view helpful or very helpful in approximately 20% of the cases. Furthermore, the reviewers had 100% reproducibility of interpretation of frontal plus lateral radiographs versus 96% for frontal radiographs alone. Surprisingly, the most experienced reviewer gave a higher score than the junior reviewers for “helpfulness of lateral view”; they were also the least likely to change their assessment with the addition of the lateral view. This could be due to differences in opinion regarding what is helpful or due to the meticulousness of the reviewer regarding interpretations and measurements (Table 1). Further study is needed to assess whether lateral radiographs should replace frontal radiographs as the standard procedure in resource-limited NICUs to support sick neonates and to avoid extra radiation exposure.

As with the majority of research, our study has several limitations. First, the study design as retrospective carries a potential for feebleness. Second, there is a lack of clinical outcome correlation with UVC repositioning after obtaining lateral radiographs. Moreover, the risk of radiation and the cost of lateral radiographs should be evaluated on the scale of the value of lateral radiographs. Finally, bedside ultrasonography is becoming the investigation of choice for optimal UCV placement in many NICUs across the world replacing routine radiographs.

## Conclusions

Our study suggests that lateral radiographs are a helpful tool in determining UVC tip position, particularly for UVCs placed at or below the level of the diaphragm. Furthermore, the addition of a lateral view to the standard frontal chest radiograph may be beneficial for the evaluation of the UVC position if a real-time ultrasound cannot be performed before UVC usage. This addition of the lateral views would be especially beneficial for the on-call junior staff.
